# Dynamic Hippocampal and Prefrontal Contributions to Memory Processes and Representations Blur the Boundaries of Traditional Cognitive Domains

**DOI:** 10.3390/brainsci7070082

**Published:** 2017-07-12

**Authors:** Rachael D. Rubin, Hillary Schwarb, Heather D. Lucas, Michael R. Dulas, Neal J. Cohen

**Affiliations:** 1 Beckman Institute for Advanced Science and Technology, University of Illinois at Urbana-Champaign, Urbana, IL 61801, USA; hschwarb@gmail.com (H.S.); hdlucas@gmail.com (H.D.L.); duulaas@gmail.com (M.R.D.); njc@illinois.edu (N.J.C.); 2 Department of Psychology, University of Illinois at Urbana-Champaign, Champaign, IL 61820, USA; 3 Carle Neuroscience Institute, Carle Foundation Hospital, Urbana, IL 61801, USA; 4 Interdisciplinary Health Sciences Institute, University of Illinois at Urbana-Champaign, Urbana, IL 61801, USA

**Keywords:** hippocampus, prefrontal cortex, memory, patient studies, brain networks

## Abstract

The hippocampus has long been known to be a critical component of the memory system involved in the formation and use of long-term declarative memory. However, recent findings have revealed that the reach of hippocampal contributions extends to a variety of domains and tasks that require the flexible use of cognitive and social behavior, including domains traditionally linked to prefrontal cortex (PFC), such as decision-making. In addition, the prefrontal cortex (PFC) has gained traction as a necessary part of the memory system. These findings challenge the conventional characterizations of hippocampus and PFC as being circumscribed to traditional cognitive domains. Here, we emphasize that the ability to parsimoniously account for the breadth of hippocampal and PFC contributions to behavior, in terms of memory function and beyond, requires theoretical advances in our understanding of their characteristic processing features *and* mental representations. Notably, several literatures exist that touch upon this issue, but have remained disjointed because of methodological differences that necessarily limit the scope of inquiry, as well as the somewhat artificial boundaries that have been historically imposed between domains of cognition. In particular, this article focuses on the contribution of relational memory theory as an example of a framework that describes both the representations and processes supported by the hippocampus, and further elucidates the role of the hippocampal–PFC network to a variety of behaviors.

## 1. Introduction

Over the past several decades, cognitive neuroscience has greatly improved our understanding of the brain systems involved in human memory, in terms of both the contributions of specific regions and the interactions among them. This increased understanding of the brain has, in turn, contributed to the ways in which memory itself is conceptualized. For instance, early studies of patients with localized brain injury led to the somewhat reductionist assumption that different regions of the brain could be directly mapped onto distinct forms of cognition (e.g., memory vs. language vs. attention vs. executive function). These early ideas about domain-specific structure-function relationships have been supported, but also challenged, by advances in other methods that provide a network- or systems-level view of how the brain operates.

In terms of memory, a vast literature highlights the importance of the medial temporal lobes (MTL), specifically the hippocampus, in creating new, long-term declarative memories [1,2,3]. Yet, there is also a continually growing body of work that suggests the hippocampus, across nearly all timescales, contributes to adaptively and flexibly guiding a wide range of behaviors, including some that have traditionally been thought to be outside the domain of memory entirely (see [4] for review).

Similarly, the prefrontal cortex (PFC) has traditionally been linked to executive function and decision-making [5], but is now known to contribute to a much wider range of behaviors, many of which are also associated with contributions from the hippocampus. For instance, PFC has gained traction as a necessary part of the memory system, which also blurs the boundaries between circumscribed domains of cognition.

Thus, the goal of this article is to synthesize these largely disjointed literatures and move towards a more parsimonious and comprehensive account of the evidence that links the hippocampus and PFC to a repertoire of abilities that have traditionally been viewed as neurocognitively dissociable. In doing so, we propose that theoretical advances are required for an improved understanding of the characteristic *representations* and *processes* supported by the hippocampus and PFC. We define representations as the structured organization of information as it exists in our mind, and processes as the manner in which that information is internally operated on and/or manipulated. Both are hypothesized as properties of the brain necessary to reflect and guide our experience; as such, constructing theories that honor both of these properties is necessary to capture the complexity of mind–brain relationships within and beyond the domain of memory.

This review begins with a brief history of landmark hippocampal and PFC patient studies. We then examine ways in which early theories, particularly with regard to hippocampus, were advanced, resulting in the development of a relatively comprehensive framework of hippocampal function that accounts for both the processes it enacts and the representations it supports. However, the process of developing and testing this framework also had the effect of blurring clear boundaries between domains linked to the hippocampus and those more typically associated with the PFC, such as memory and decision-making, respectively. To better understand this observation, we briefly review current PFC theories and hippocampal–PFC network interactions. In particular, we focus on evidence that the representations and processes supported by the hippocampus and PFC interact in an iterative manner, blurring the lines between encoding and retrieval in memory, and facilitating the performance of a wide range of behaviors outside of memory. Lastly, we identify that specific theoretical advances are required in order to continue making progress toward accounting for the breadth of findings that illuminate the contributions of hippocampus and PFC to human behavior.

## 2. Patient Lesion Studies Initially Identified Distinct Contributions of Hippocampus and PFC to Different Domains of Cognition

Early attempts at characterizing brain function relied primarily on patient lesion studies to assess the relationship between focal brain damage and performance on various tasks. This method of investigation allows researchers to infer that deficits in performance are causally attributable to the damaged region, such that the region is necessary to perform tasks in a particular domain successfully. The strongest evidence comes from studies that identify double-dissociations between patients with damage to different regions of the brain and subsequent impairments on tasks in one domain, but not in the other, and vice versa.

For instance, our understanding of the contribution of the MTL, and in particular the hippocampus, to memory originated from famous studies with patient H.M. and others like him that had relatively circumscribed MTL damage. These patients were severely impaired in their ability to form new long-term declarative memories, while other domains of cognition (i.e., intellect, language, executive function) and other kinds of memory (i.e., procedural memory, short-term and working memory) initially seemed to remain intact [6,7,8]. These other domains were found to rely more heavily on other brain structures, adding support for the functional specialization of different regions to traditional cognitive domains.

Similarly, early insight into PFC function resulted from the famous example of Phineas Gage, who reportedly experienced profound executive function impairments and personality changes after an isolated injury to the PFC, causing him to become more impulsive, aggressive, agitated, and seemingly less capable of the ability to exert control over his own behavior and decision-making capacity [9]. Although initial accounts of these changes may be exaggerated, there are a number of recent findings consistent with this report that attribute PFC damage to severe behavioral dysregulation and impairments in executive function and decision-making abilities [10,11].

These early studies were pioneering in conceptualizing structure–function relationships in the brain. Nonetheless, our theories must continue to reflect and integrate advances from multiple methodologies. That is, we must seek to account for new data from converging methods, and continually assess whether the accumulation of data about the organization of brain function upholds the conventional boundaries between cognitive domains. Indeed, as discussed in the next section, one barrier to achieving a parsimonious view of hippocampal function has been that human and animal studies historically emphasize different aspects of its function, and recent attempts to reconcile these literatures has resulted in considerable progress toward developing a comprehensive theory of hippocampal function.

## 3. Hippocampal Contributions Extend beyond the Historically Circumscribed Domain of Memory

As previously mentioned, traditional views of hippocampal function in humans highlight its critical role in the formation and conscious recollection of long-term declarative memories (e.g., [1,6,12]), whereas the animal literature emphasizes the importance of the hippocampus for spatial cognition and navigation (e.g., [13,14]). Similarities and differences between long-term memory and spatial processing accounts of the hippocampus have been reviewed elsewhere [15]. However, it bears mentioning that each of these literatures produced key insights that challenged supposed constraints on the reach of hippocampal processing suggested by the other. For example, theories that emphasize spatial cognition have traditionally imposed the constraint that the *representations* supported by the hippocampus are limited to those that are spatial in nature. By contrast, extensive research from human subjects illustrates that any type of information—be it spatial, temporal, social, or otherwise—can be represented by the hippocampus as long as it is relational in nature [1,2,4,16,17,18,19,20,21,22,23,24,25,26,27,28,29,30].

Conversely, work in animals linking the hippocampus to spatial navigation has challenged the notion that the *processes* afforded by the hippocampus are limited to those contributing to the ability to learn and retain information across long delays. Particularly illustrative is the body of work demonstrating the interactive roles of the hippocampus and PFC in online decision-making (i.e., in the moment, during ongoing processing), as it recognizes the contribution of hippocampal representations and processes to both a domain and timescale previously only associated in humans with PFC. This work provides evidence from animals and humans that the hippocampus contributes to decision-making behavior, including actively foraging or exploring within an environment to gather and weigh the information necessary to inform immediately upcoming behaviors [31,32,33,34,35,36]. For example, at choice-points during exploration (e.g., at the intersections of a T maze), activity in rodent hippocampal cells alternates between representations of the two possible endpoints, permitting “vicarious” exploration of potential outcomes [37,38].

In sum, decades of evidence from animal studies, human patient studies, and neuroimaging is now converging to indicate that the reach of the hippocampus extends much further than previously appreciated (for review, see [4]). This work shows that (1) the hippocampus contributes to memory performance across all timescales, including short-term/working memory [39,40,41,42] and on-line processing (e.g., [36,43,44,45,46,47]), and (2) the hippocampus also contributes to performance on tasks considered to be outside the domain of memory, such as language use [23,48,49,50,51], imagination [52,53,54,55,56], creativity [16,57], empathy [58,59], character judgments [26,60], decision-making [61,62,63,64], and problem solving [57]. Further, consistent with the animal work mentioned above, human studies have provided corroborating evidence that the hippocampus is important for spatial navigation [21,65,66,67]. Lastly, there is evidence that remote sematic memory is impoverished in hippocampal amnesia [68], which we discuss more later on, as the finding stands contrary to many accounts that posit remote semantic memory is independent of the hippocampus [69,70,71].

It is worth noting that many of the above discoveries came from studies of patients with focal brain lesions. That is, the same method that was initially used to generate a long-term memory centric view of hippocampal function has, in recent years, been instrumental in shedding light on hippocampal contributions across domains, and continued insight is still likely to come from the unique contributions of patient studies [72,73]. Thus, we argue that theoretical advances, as opposed to purely methodological or technical achievements, are instrumental in moving towards a more comprehensive account of hippocampal and PFC function.

## 4. Relational Memory Theory Describes Both Memory Processes and Representations Supported by the Hippocampus

Relational memory theory [1,2,15,18] has played a significant role in driving much of the research described above, expanding our appreciation of hippocampal contributions to a broad range of cognitive and social behaviors, as well as laying the foundation for a more nuanced understanding of the dynamic interaction between hippocampal and PFC regions in memory and beyond. We argue this is precisely because relational memory theory offers a view of hippocampal function that accounts for its role in both memory processes *and* representations.

Here, we provide the core tenets of relational memory theory, which are developed in detail in the original proposal [1,2]. The hippocampus (1) supports the *process* of binding together arbitrarily co-occurring elements of experience into a compositional *representation* (i.e., the bound configuration of the relations between the elements of experience). The elements of experience may include the people, places, and things, along with the spatial, temporal, and interactional relations among them. Further, the hippocampus (2) permits the flexible activation and reactivation of such representations in response to a wide variety of task demands, thereby uniquely enabling the system to make use of elements that were “only” arbitrarily related at one time.

In terms of arbitrary relations, we mean the co-occurrence of elements with no inherent a priori relationship, such as the name associated with a face or the specific digits that make up a phone number. Notably, it is often the case that the significance of arbitrarily related elements of experience can only be appreciated later, and flexibly reassembled for different means, at different times, over the course of one’s life (e.g., you only later find out the person that sat at the table next to you in the café is also a hippocampal enthusiast and so you eagerly look for them in the same spot, at the same time tomorrow). This function is uniquely attributed to the hippocampus.

As such, relational memory theory is notable in that it describes hippocampal contributions to memory in terms of the *processes* supported by the hippocampus (i.e., arbitrary binding and later reactivation), as well as the nature of hippocampal *representations* (i.e., compositional/relational). Relational memory theory has advanced the theoretical characterization of hippocampal function and provided a more nuanced understanding of its contributions to declarative memory, which was previously limited to memory that was only “explicitly” stated or consciously “declared” [74]. It provides a framework that can simultaneously account for hippocampal contributions to declarative memory, as well as to behaviors not traditionally viewed as memory behaviors per se, but which require the same kind of characteristic processes and representations. Thus, it is the manner by which relational memory theory describes characteristic hippocampal processes *and* representations that enables the synthesis of seemingly disparate findings in the literature.

## 5. PFC Contributions Extend beyond Historically Circumscribed Domains and Include Memory

The myriad of cognitive functions currently ascribed to PFC and its various subregions are diverse and extensive, reaching well beyond executive function and decision-making. Thus, a complete review of the robust literatures on the localization of PFC function to its subregions is outside the scope of this paper (for reviews, see [5,75,76,77,78]); however, we aim here to provide a survey of the findings that highlight behaviors where PFC and hippocampal contributions are each recognized as important, and acknowledge the contributions of specific PFC subregions from particular studies where appropriate. For instance, creativity, empathy, and problem-solving are cognitive and social behaviors for which the role of PFC is commonly recognized, though contributions from hippocampus have recently been noted (as described above). Indeed, patients with frontal pole damage are typically impaired on standardized tasks of creative thinking (e.g., Torrance Test of Creative Thinking [79]) and patients with lateral PFC damage are impaired on tasks of divergent thinking [80]. Diminished empathy is also a hallmark feature of PFC damage [58]. Likewise, successful problem-solving often relies on successful prediction of action outcomes, which is supported by ventromedial PFC structures [81], or on successful abstract rule extraction/use supported by lateral PFC structures [82].

PFC also contributes to both working memory and episodic memory tasks. The dorsolateral PFC (dlPFC), in particular, is thought to play an essential role in working memory processes [83,84,85]. In support of this view, single-cell recording work demonstrates that dlPFC neurons fire selectively during the delay period of a working memory task [86], and human patients with dlPFC lesions are significantly impaired on short-delay working memory tasks [87]. Further, on episodic memory tasks, dlPFC has been shown to contribute by exerting control over retrieval processes and monitoring the accuracy of retrieved information required for successful memory performance [88,89,90,91,92,93,94]. For instance, patients with dlPFC damage are grossly impaired on tasks that require controlled associations of remembered information (e.g., A–B, A–C tasks) [95]. Similarly, rodents show memory deficits on tasks requiring a switch between subsets of remembered information when medial PFC is damaged [96].

Varying accounts of PFC contributions to behavior often appear disjointed, in part because the reach of PFC is so extensive and its anatomical landscape comprises many distinct subregions. In an effort to provide a cohesive account of PFC function, multiple theories have been developed that provide important organizational insights for understanding the diversity of PFC *processes* (for reviews, see [5,75,76,77,78]). We suggest that this process-centric approach may necessarily limit our full ability to understand the range of PFC contributions to behavior because so little theoretical attention is given to the nature of PFC representations. As our understanding of hippocampal function benefited from a theoretical framework that considered both processes and representations (i.e., relational memory theory), we suggest PFC theories would likewise benefit from the consideration of both aspects of brain function. Such a “hybrid” approach may help account for the similarities in behaviors where PFC and hippocampal contributions are each recognized as important. Further, in line with recent experimental and theoretical work, a hybrid approach focuses on the hippocampus and PFC not as independent entities, but rather as part of a functionally and structurally connected network of brain regions.

## 6. Hippocampal–PFC Network Interactions Disregard the Boundaries of Traditional Cognitive Domains

The importance of hippocampal–PFC networks to cognition is increasingly recognized, but often discussed either in terms of their contribution to memory, or more recently, their role in decision-making, domains that were historically associated with the hippocampus or PFC, respectively. In line with the traditional conceptualization of hippocampal function, hippocampal–PFC network contributions were initially identified for long-term memory, noting the role of the hippocampus in memory encoding, storage, and retrieval, and the PFC in cognitive control processes [97], including selection [92,93,94], engagement of retrieval mode [98], monitoring [91,99], and inhibition [100]. Other work noted that the same PFC regions contributed to both long-term and working memory [101,102,103] tasks, but often neglected the contribution of hippocampus to tasks outside of long-term memory.

A number of studies, however, demonstrate hippocampal–PFC network interactions across these previously circumscribed domains and timescales. Replay in hippocampal cell ensembles is believed to contribute to memory consolidation [104], so that memory for past experiences can inform current behavior. Importantly, activity in these hippocampal cells also drives activity in PFC cell ensembles [105,106]. This phenomenon has been attributed to evaluating/executing possible decisions and/or abstracting “rules” about the environment [107]. Strong evidence from human neuroimaging studies also suggests that adaptive exploration and online decision-making involve bidirectional communication between the hippocampus and PFC as part of a coordinated functional network [34,35,108,109,110,111].

Structural connections between the hippocampus and PFC likely facilitate this coordination. The uncinate fasciculus is a white matter tract that connects the anterior temporal lobe to the orbitofrontal cortex and passes adjacent to the hippocampus [112]. Recently, the role of uncinate fasciculus integrity in memory-guided decision-making was evaluated in a task designed to rely on contributions from both the hippocampus and PFC. The task was a relational memory task that also incorporated implicit, abstract rules, which could be learned across trials to facilitate performance [113,114]. Responses were made based on a combination of relational memory and implicit rule use. That is, the decision could be made using both specific information from the episode and abstract information gathered across episodes. Interestingly, uncinate fasciculus integrity correlated with successful rule use for both studied and novel stimuli, highlighting the contribution of hippocampal–PFC interactions in memory-guided decision-making.

Findings such as these suggest that the integration of the memory and decision-making literatures is essential in moving towards a comprehensive account of the full range of hippocampal–PFC network contributions. In the next section, we elaborate on the processes and representations supported by the network. While our perspective considers hippocampal processes and representations in terms of relational memory theory, we recognize the efforts of others in generating productive theories of PFC function, which when taken together, serve to further elucidate the breadth of hippocampal–PFC contributions.

## 7. Theories of Hippocampal–PFC Networks Must Account for the Dynamic Interaction of Both Processes and Representations

We have presented relational memory theory as an account of hippocampal *processes* and *representations* that contribute to various cognitive and social behaviors. By contrast, theories of PFC tend to focus more exclusively on *processes* [115], perhaps attributable to the traditionally recognized role of PFC in executive functioning and decision-making, noting that the *–ing* itself implies some form of action. Indeed, there is strong evidence from functional neuroimaging that PFC engages in operations that guide, organize, and/or modify representations maintained by other regions. This type of processing is evident both when information is encoded, by providing top-down modulation of representations in the visual cortex (e.g., to enhance attended-to information and to suppress to-be-ignored information [116,117,118]), as well as when information is retrieved (e.g., to reduce reactivation of irrelevant information [119]).

However, evidence also exists that certain PFC subregions may be essential in maintaining information that cannot be represented elsewhere [115]. For instance, patients with lesions to ventromedial PFC (vmPFC) demonstrate behavior that reflects diminished schematic representations; both when the integration of newly learned valenced-information is required, as in updating character judgments [26], as well as when the organization of remote semantic information impacts responses, as on the Deese–Roediger–McDermott (DRM) paradigm [120]. In these cases, patients with vmPFC lesions appear to have deficits in creating and using integrated, abstract representations to guide behavior. The suggestion then is that these abstract representations, or schemas, may indeed be stored in this region, and that they are continually shaped, updated, and modified via hippocampal–PFC interactions in the intact brain. These works and others recognize similarities in the contributions of vmPFC to schemas, memory, and decision-making [121,122]. Lateral PFC regions have also been linked to the generation and evaluation of action plans during decision-making [123], though the extent to which action plan representations are supported, rather than merely acted upon, by lateral PFC regions remains unclear.

Thus, in attempting to understand the contributions of the hippocampal–PFC network to behaviors across domains, it will be fruitful for future studies to generate and test hypotheses that jointly describe both the processes and representations supported by each brain region. While this has not yet fully been achieved, there has been notable progress. For instance, there are hippocampal–PFC network theories that suggest the hippocampus is essential for creating memory representations, while the PFC is responsible for processes that involve switching between remembered information to bias retrieval of task-relevant information [77]. However, these theories do not detail the nature of hippocampal representations and processes, or PFC representations.

Other theories, which primarily emphasize hippocampal–PFC contributions to memory, do incorporate the nature of hippocampal processes and representations, building upon ideas put forth by relational memory theory. In particular, they suggest the hippocampus binds together arbitrarily co-occurring elements of experience (i.e., arbitrary relations) into a compositional representation, while areas of PFC build up more inherently meaningful relations, abstracting representations of the larger “context” in which specific memories take place (e.g., a list in which specific words appear, or a room in which a certain set of experiences tend to occur), thus aiding in context-dependent memory retrieval [88,113,124]. It has also been demonstrated that hippocampal–PFC contributions to memory formation and its use are iterative, such that: (1) new memories are integrated within existing schemas, and (2) existing schemas are further modified in the process of memory consolidation [88,125]. As such, these theories offer more in terms of how PFC processes interact with hippocampal processes and representations, but it is still not clear to what extent context-dependent processing (i.e., retrieval) influences the nature of PFC representations, or how it makes contact with other domains of cognition, such as decision-making.

Consideration of the above ideas has led to an extension of relational memory theory in the form of a recently proposed framework—termed Covert Rapid Action-Memory Simulation (CRAMS) [126]—that underscores the online coordination of hippocampal–PFC network interactions as critical to exploratory behaviors (i.e., navigation) and memory-guided decision-making. This framework builds on relational memory theory, discussing hippocampal processes and representations, while also fleshing out the coordinated involvement of PFC in (1) context- or rule-guided behavior (e.g., extracting and using statistical regularities in the environment), and (2) the planning and execution of complex actions (e.g., action planning). According to the CRAMS framework, the hippocampus automatically and obligatorily binds relational information, enabling access to a body of knowledge that is potentially relevant to upcoming decisions, which PFC consults in the process of generating/simulating (lateral PFC) and evaluating (medial PFC) potential action plans. This network then engages in the rapid, iterative, and at times covert, exchange of information, allowing the selection of the most advantageous choice in a given situation. Although this framework also does not fully describe the nature of PFC representations, it establishes the contribution of the hippocampus and PFC, via their network properties, to both memory and decision-making, hypothesizing the iterative interactions of this network over time to guide future behavior. Given the role of PFC in schemas and task-sets [120,122,125,127,128,129,130,131], it is likely that PFC-mediated representations also serve to guide decisions. That is, a particular choice/response conflict is likely to have some similarity to a schema used for previously encountered decisions, allowing PFC representations to potentially guide the proposed specific, covert memory retrieval of the hippocampus.

Interestingly, the nature of hippocampal–PFC network interactions proposed by CRAMS may also underlie the potential mechanism for the creation of hippocampally-independent representations (i.e., the manner in which newly learned information is eventually consolidated into long-term memory). This notion is in line with the complementary learning systems framework [132,133], in that the hippocampus is responsible for quickly acquiring rich, distinct memory representations, while the neocortex (in this case the PFC) extracts and integrates information over time to create broader schematic representations, via iterative interactions with the hippocampus. As noted previously, making decisions and encoding new information do not occur in isolation, but rather both are informed by previously learned information and occur in the context of existing schemas, which in turn allow representations to be iteratively updated. Thus, the interplay between not only hippocampal and PFC processes, but also hippocampal and PFC representations, allows even well-consolidated semantic concepts to be updated and enriched with new information. This proposal provides an explanation of the finding that, contrary to conventional accounts, remote sematic memory is impoverished in hippocampal amnesia [68]. While patients with hippocampal amnesia perform in the normal range on superficial tests of semantic memory (e.g., word-definition matching), they show impairments on measures of semantic memory depth and richness. That is, remote semantic memory representations may functionally exist independently of the hippocampus, but damage to the hippocampus prevents them from being perpetually and iteratively shaped by new experiences. Moreover, without this updating, existing remote semantic representations may not acquire novel connections between each other, leading to the impaired ability to flexibly relate even previously learned representations or to use/apply previously learned schemas to novel situations.

It is important to note that by invoking both representations and processes of the PFC and hippocampus, as well as their iterative interplay, the concepts of separable encoding and retrieval “phases” of memory become less distinct. For example, it may be that during the study phase of a memory experiment, the retrieval of existing PFC representations serves to guide what should be bound in memory during encoding, and the rapid and iterative interaction between PFC and MTL during this “phase” involves multiple instances of encoding and retrieval, even at the level of a single trial. Similarly, during the test phase, retrieval is not occurring in isolation; rather re-encoding occurs as we retrieve information, potentially impacting and altering the contents of memory both at the hippocampal level (i.e., distinct episodic representations) and potentially in the neocortex (e.g., subtle changes to schema representations in the PFC) [134]. Also, while CRAMS discriminates between medial PFC-mediated evaluation processes and lateral PFC-mediated simulation processes, it does not fully address whether these PFC regions also store distinct types of representations.

**Figure 1:**
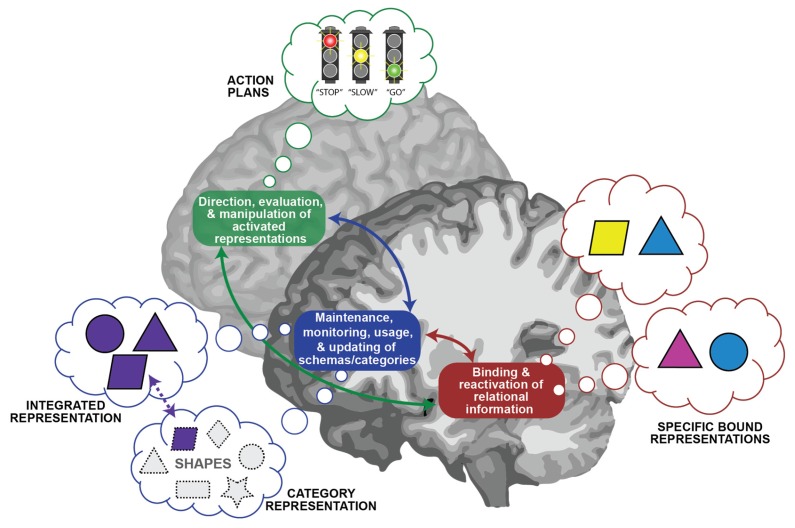
Hippocampal–Prefrontal Cortex (PFC) Network Processes and Representations. Boxes = processes; Thought bubbles = representations; Red box and thought bubbles = mediated by hippocampus; Blue box and thought bubbles = mediated by medial PFC; Green box and thought bubble = mediated by lateral PFC. In this example, the hippocampus supports processes and representations consistent with relational memory theory, permitting the arbitrary binding and later reactivation of various attributes of the event (e.g., spatial, temporal, and associative relations, such as here, which shapes were present and how they were arranged relative to each other). The medial PFC supports the maintenance, monitoring, usage, and updating of more abstract or meaningful information, whereas the lateral PFC supports the direction, evaluation, and manipulation of this information. We suggest medial PFC regions represent information about abstract categories that are related to the elements (in this case, abstract “shape” categories such as stars, rectangles, etc.), as well as integrated representations of the elements across the events (e.g., the combination of shapes in the events, but with less detail of their arrangement relative to each other). We also suggest lateral PFC may maintain representations of action plans and, like a stop signal, inhibit, slow down, or activate/enhance, the simulation of behaviors required to perform a task (e.g., in this case, deciding to initiate movement of the shapes to achieve a particular goal). Further, the processes naturally unfold over time, allowing the representations to be iteratively shaped (i.e., modified, updated) by new experiences.

Therefore, in Figure 1, we elaborate upon the medial and lateral PFC-mediated processes outlined by CRAMS to demonstrate how possible PFC representations may be included in a framework that incorporates both processes and representations supported by the hippocampal–PFC network. This is based on evidence that links medial PFC to abstract category and integrated representations [26,120,135,136], as well as evidence that lateral PFC acts upon active representations, such as action plans, facilitating the integration of information, although it is less clear if lateral PFC also maintains these representations long-term [75,82,123,137,138,139,140]. CRAMS [126], and others [121,122], identify medial PFC as the site of evaluation/monitoring, but there is evidence implicating the lateral PFC in monitoring processes as well [91,141,142,143]. We propose medial PFC monitoring may be tied more to confidence, schematic instantiation, and valuation signals [121,136,144,145,146], whereas the lateral PFC may be more involved in evaluating, comparing, and selecting between multiple representations [91,94,99,137,147,148,149,150,151]. However, more work is needed to disentangle the distinct types of monitoring that the medial and lateral PFC distinctively support.

Critically, our proposed framework is useful in that it identifies properties of the network that are relevant across tasks; it can be applied to the role of the network in long-term memory (as in the example in Figure 1), as well as to its role in online processing in other domains. For example, consider the case of generating creative uses for cardboard boxes (i.e., from the standardized test of creativity—the Torrance Test of Creative Thinking—used in our work with hippocampal patients [16]). In this task, category representations and action plans to simulate possible uses (e.g., storage uses, artistic uses, fun/play uses), via PFC, serve to guide the generation of specific examples (e.g., the arrangement of stacked boxes, the creation of a sculpture, the building a fort), via hippocampus, and then these representations can be iteratively processed across the network, allowing novel “uses” to be evaluated in comparison to an already generated list.

Likewise, while CRAMS was initially conceived as a framework for memory-guided decision-making, its ideas and those proposed here may help explain the breadth of behaviors supported by the hippocampal–PFC network. That is, although online decision-making has been mostly described in terms of exploration via spatial navigation, it need not be limited to it. Decision-making often involves the “exploration” of environments that are non-physical. This includes word-finding (i.e., searching lexical/semantic space) and creative problem solving (i.e., searching for flexible relationships among semantic concepts). Indeed, evidence linking PFC to the executive control of language [152,153,154], along with the previous examples of hippocampal contributions to language and creativity [23,49,50,155,156], provide support for this notion. Thus, we submit that the iterative interaction between hippocampus and PFC serves in the creation, updating, and utilization of representations important for all of these behaviors, by iteratively integrating the more meaningful, abstract rule sets and schemas of the PFC, with the more arbitrary and distinct relational representations of the hippocampus, and vice versa.

## 8. Conclusions and Future Directions

This review emphasizes that the ability to parsimoniously account for the breadth of hippocampal and PFC contributions to memory function and beyond requires theoretical advances in our understanding of the characteristic processing features *and* mental representations supported by the hippocampus and PFC. That is, theoretical advances must account for the growing amount of evidence that blurs the contributions of particular regions across domains.

We focused on the contribution of relational memory theory, and its extension, CRAMS, as examples of frameworks that describe both the representations and processes supported by the hippocampus and the role of the hippocampal–PFC network in a variety of behaviors across historically circumscribed domains. Further, we proposed that this framework, in conjunction with evidence of PFC-mediated representations, may be extended to potentially account for the wide range of behaviors the hippocampal–PFC network supports. Future research should include theories that characterize both the nature of PFC representations *and* processes, in order to more fully appreciate the dynamic contributions of brain networks, such as the hippocampal–PFC network, to the full breadth of sophisticated human behaviors.

Finally, it is worth recognizing that progress aligning our understanding of cognition with brain networks has direct clinical implications, including the ability to better understand and treat disorders of mental health and brain injury [157,158]. Similar ideas have been put forth by the National Institute of Mental Health (NIMH). Notably, the Research Domain Criteria (RDoC) project encourages researchers to shift their focus to disruptions of dimensions of cognition and behavior, or underlying symptoms, instead of the categorically distinct mental disorders currently described in the Diagnostic and Statistical Manual of Mental Disorders (DSM-5). Likewise, clinical researchers have aimed to bridge the gap between clinical and cognitive approaches where shared mechanisms are hypothesized, e.g., relating particular forms of psychopathology and impairments of executive function [159], which may also share disruptions to the hippocampal–PFC network based on the findings presented here. These proposals are alike in that historical boundaries between domains are being challenged based upon the functional and structural properties of the brain.

Thus, we look forward to theoretical advances that aim to broadly cut across literatures and data from diverse methodologies in order to improve our understanding of human memory and more fully develop its contribution to other areas of cognition and social behavior.
